# Multiple imputation with compatibility for high-dimensional data

**DOI:** 10.1371/journal.pone.0254112

**Published:** 2021-07-08

**Authors:** Faisal Maqbool Zahid, Shahla Faisal, Christian Heumann

**Affiliations:** 1 Department of Statistics, Government College University Faisalabad, Faisalabad, Pakistan; 2 Department of Statistics, Ludwig Maximilians University Munich Germany, Munich, Germany; Vellore Institute of Technology: VIT University, INDIA

## Abstract

Multiple Imputation (MI) is always challenging in high dimensional settings. The imputation model with some selected number of predictors can be incompatible with the analysis model leading to inconsistent and biased estimates. Although compatibility in such cases may not be achieved, but one can obtain consistent and unbiased estimates using a semi-compatible imputation model. We propose to relax the lasso penalty for selecting a large set of variables (at most n). The substantive model that also uses some formal variable selection procedure in high-dimensional structures is then expected to be nested in this imputation model. The resulting imputation model will be semi-compatible with high probability. The likelihood estimates can be unstable and can face the convergence issues as the number of variables becomes nearly as large as the sample size. To address these issues, we further propose to use a ridge penalty for obtaining the posterior distribution of the parameters based on the observed data. The proposed technique is compared with the standard MI software and MI techniques available for high-dimensional data in simulation studies and a real life dataset. Our results exhibit the superiority of the proposed approach to the existing MI approaches while addressing the compatibility issue.

## 1 Introduction

Missing data are frequently encountered in biomedical research. The statistical analysis of the data often demands complete cases without any missing values. The analysis without appropriate handling of missing values may lead to biased inferences. A variety of statistical methods is available for addressing the missing data issue. Multiple imputation (MI) [1–3] has become the most popular approach for handling missing data in practice. MI fills each missing value with more than one plausible value drawn from its predictive distribution given the observed data. As a result, we have several imputed datasets that account for the uncertainty due to the imputation process. MI formally comprises three stages: *imputation*, *analysis*, and *combining results* of the analysis. In the *imputation stage*, *M* independent imputed values are obtained corresponding to each missing value to get *M* complete imputed datasets. In the *analysis* stage, each of the *M* imputed datasets is analyzed using standard statistical techniques for complete data. In the third stage, *M* sets of desired estimates are combined into one set of parameter estimates using Rubin’s rules [1].

Sequential regression [4], also known as chained equations [5, 6] or Fully Conditional Specification (FCS) [7], is the usual approach to obtain multiply imputed data. This approach imputes the data on a variable-by-variable basis by specifying an imputation model for each missing variable given the other variables. The available regression based methods for multiple imputation rely on fitting the imputation model using Generalized Linear Models (GLM). The use of GLM limits the number of predictors with respect to the sample size. It will not be an exaggerated claim that MI has become the most popular approach in the recent years due to its flexibility and advancement in the methodology and software. But for missing values in high-dimensional data (*p* > *n* or *p* ≫ *n*), MI may confront different issues during the imputation. First, which and how many variables should be included in the imputation model to make GLM fit possible. Second, the standard implementations of MI usually assume that the data are missing at random (MAR). Although MAR is a non-testable assumption, one hopes to get very close to MAR if enough variables are included in the imputation model. Mostly, MI is used directly by relying that the data fulfills the MAR assumption. Third, it is always recommended to include as many variables as possible in the imputation model [8]. The MAR assumption tends to be more justifiable as more variables are added to the imputation model. But on the other hand, likelihood estimates of the imputation model may become unstable as *p* → *n*. Fourth, the imputation model should be compatible (or at least semi-compatible) with the substantive model [9, 10]. Compatibility is related to Meng’s concept of congeniality [11], and the term congeniality is often used to mean compatibility [5, 12]. Two conditional densities are compatible if a joint distribution exists that has the given densities as its conditional densities. An imputation model will be semi-compatible, if a restricted version of it is compatible with the substantive model. The incompatible imputation model may lead to inconsistent and biased parameter estimates during the analysis stage (discussed in the following text).

There is no ultimate strategy available to decide the number and choice of predictors in an imputation model when *p* > *n*. In case of many potential variables, a selection becomes inevitable using some formal variable selection procedures. There are some classical methods like stepwise regression which are more popular among non-statisticians. The problem with stepwise regression is that it fits the model using unconstrained least squares at any given step. A better option is to use the approaches that group or constrain the coefficient estimates in some way, e.g., lasso (least absolute shrinkage and selection operator) proposed by [13]. Following lasso, many variable selection methods based on penalized likelihood have been developed in the literature. [14] used penalization for the selection of variables in the imputation model. But the plausibility of the MAR assumption, and the compatibility of such an imputation model can be questionable. We advocate the generally accepted rule for building the imputation model in MI that it should be as general as possible.

To address the above mentioned issues, we propound to build a rich imputation model by accommodating a maximum number of potential candidate predictors. Our idea is to use L1 penalty for accommodating the maximum number of candidate predictors (at most *n*) in the imputation model, and fitting the resulting model using L2 penalty. We expect the substantive model based on some formal variable selection approach to be nested in our rich imputation model. The likelihood estimation for a big model can be problematic. As *p* → *n*, the likelihood estimates may not converge, and if they converge, the obtained parameter estimates may have infinitely large standard errors. The use of ridge regression for the selected rich imputation model will guarantee the unique parameter estimates and will lead to better predictions with a good compromise between bias and variance. The relaxing of lasso penalty to select an imputation model with maximum number of predictors assures that: (i) the MAR assumption will become more plausible, (ii) the set of variables that can be selected using the optimal value of L1 penalty found by cross-validation will also be present in our imputation model, and (iii) there will be high chances of achieving semi-compatibility necessary to get consistent and unbiased estimates. The proposed setup is also suitable for *n* > *p* situations where the imputation model can be fitted without performing any selection of variables.

## 2 Penalization for variable selection and model fitting

Penalization is a natural choice for fitting the imputation model in high-dimensional data structures where many potential candidate predictors are available. In this Section, we briefly review the regression with L1 and L2 penalties. For the sake of simplicity we use the same notation $\hat{\beta }$ for likelihood estimates and penalized estimates of the regression parameters ***β***. However, wherever needed, the approach used for estimating ***β*** is stated explicitly.

### 2.1 Regression using ridge penalty

Hoerl and Kennard [15] proposed ridge regression for multiple linear regression as a solution to non-orthogonal problems. In the GLM context, [16, 17] described ridge estimates for logistic regression, and [18, 19] extended the ridge regression for GLMs. Let **X** = (**x**_1_, …, **x**_*p*_) be a *n* × *p*-dimensional data matrix in a GLM. The mean *μ*_*i*_ is related to the linear predictor $\eta_{i}(=x_{i}^{T}\beta )$ as *μ*_*i*_ = *h*(*η*_*i*_). Let **D** and **W**, functions of ***β*** in an iterative scheme, be *n* × *n* diagonal matrices. The *i*th diagonal element *D*_*i*_ of **D** is the derivative of the response function *h*(***η***) evaluated at *η*_*i*_ and is given by *D*_*i*_(***β***) = *dh*(*η*_*i*_)/*d**η***. Similarly, the *i*th element of the weight matrix **W** is given as $w_{i}=\sigma_{i}^{2}=\text{var}\left[h\left(\eta_{i}\right)\right]$ for *i* = 1, …, *n*. The maximization of the penalized log-likelihood $l\left(\beta \right)+\lambda \sum_{j=1}^{p}{\beta_{j}}^{2}$ (with *l*(***β***) as the log-likelihood function) will provide the ridge estimates as follows:

$$\begin{array}{ccc} \hat{\beta } & = & {\left(X^{T}WX+\lambda I\right)}^{-1}\;X^{T}WD^{-1}\left[y-\mu \right],\;\text{with}\;\text{cov}\left(\hat{\beta }\right)\\ \hat{\Sigma }\left(\hat{\beta }\right) & = & {\left(X^{T}WX+\lambda I\right)}^{-1}\left(X^{T}WX\right)\;{\left(X^{T}WX+\lambda I\right)}^{-1}, \end{array}$$ (1)

where λ ≥ 0 is a shrinkage parameter that controls the amount of shrinkage. The larger the value of λ, greater the amount of shrinkage of the parameters towards zero. For linear models, $\hat{\beta }$ and $\text{cov}(\hat{\beta })$ can be given by $\hat{\beta }={\left(X^{T}X+\lambda I_{p}\right)}^{-1}X^{T}y$ and $\hat{\Sigma }\left(\hat{\beta }\right)={\left(X^{T}X+\lambda I_{p}\right)}^{-1}X^{T}\text{var}\left(y\right)\;X\;{\left(X^{T}X+\lambda I_{p}\right)}^{-1}$ respectively.

### 2.2 Regression using lasso penalty

The lasso regression uses L1 penalty and is a popular method in high-dimensional data to achieve a sparse solution. A variety of research followed lasso to develop effective regularization methods to obtain sparse solutions. But lasso gained the more popularity and a huge amount of research used somehow the idea of lasso to develop new techniques for variable selection and model fitting [20]; [21–24]. The penalty term used by lasso is $\sum_{j=1}^{p}|\beta_{j}|$. The shrinkage parameter λ ≥ 0 controls the strength of the penalty and we get the usual likelihood estimates for λ = 0. For λ = ∞, lasso sets all parameter estimates to zero. For λ between these two extremes, the nature of L1 penalty sets some of the coefficients exactly to zero and thus doing some variable selection. As λ → ∞, not only more coefficients are set to zero (less variables are selected) but nonzero coefficients will also have more shrinkage to zero.

## 3 Proposed multiple imputation

### 3.1 Why a rich imputation model?

This Section highlights the need of a rich imputation model with more predictors along with discussing its pros and cons. In high-dimensional data structures, rich imputation models accommodating the maximum number of predictors can increase the chances of gaining semi-compatibility. The missing at random (MAR) assumption for MI becomes more rational as the number of completely observed variables is increased in the imputation model. Furthermore, the imputation model should be compatible (or semi-compatible) with the substantive model to get unbiased and consistent parameter estimates [25]. Compatibility refers to the existence of a joint model for which the corresponding conditionals are equal to both the imputation model and the analysis model. An imputation model is said to be semi-compatible if the analysis model is embedded in it. The concept of compatibility is closely related to the Meng’s concept of congeniality [11] that refers to the use of the same model for imputation and for the analysis. Both of these terms are often used alternatively.

The compatible or semi-compatible imputation models perform better than the incompatible imputation models, even when they are misspecified [26–28]. Schafer [10] supported the concept of *superefficiency* used by [8, 11]. He suggested to include a maximum number of predictors in the imputation model, even when they are not likely to be used in the substantive model, to obtain more efficient estimates. According to [29], a more restrictive imputation model from the imputer’s view point to make it consistent with God’s model (which is always unknown) can be very harmful. The imputer should be liberal in using predictors for the imputation models to help as many subsequent analyses as possible.

A problem one can face by accommodating more predictors in the imputation model, is over-fitting. The impact of such an inclusion will be at worst neutral instead of being harmful, otherwise such over-fitting will be beneficial [26]. The cost of omitting important predictors (biased estimates and invalid inferences) is often greater than the cost one has to pay (in terms of reduced precision of final estimates) when including unimportant predictors [30, 31].

### 3.2 Proposed algorithm

Let **X** = (**x**_1_, …, **x**_*p*_) be a *n* × *p*-dimensional data matrix with missing information and **y** is the completely observed response variable. If **y** has also some missing values, they will also be imputed similarly like the imputation of missing values in the covariates. Although our **y** has no missing value, it will be used in the fitting of regression model with multiply imputed datasets obtained from our proposed algorithm and other algorithms under study for comparison, as mentioned in next Sections 4.1 & 5 with corresponding results presented in Tables 2 & 4 respectively. The data matrix can be split into two mutually exclusive sets of variables i.e., partially observed covariates $X_{n\times q}^{\text{mis}}$, and completely observed covariates $X_{n\times (p-q)}^{\text{comp}}$. Let $n_{j}^{\text{mis}}$ and $n_{j}^{\text{obs}}$ be the number of missing values and observed values in the variable $x_{j}^{\text{mis}}$, respectively, *M* is the required number of multiple imputations, and $X_{m}^{\text{imp}}$ denotes the *m*th imputed dataset.

The first step of our proposed algorithm is the initialization of missing values in each $x_{j}^{\text{mis}}$. The initial filling of missing values can be done using mean imputation, *k*–nearest neighbours imputation or any other imputation method. We used, for $x_{j\;\in \{1,\ldots ,q\}}^{\text{mis}}$, a random sample from the $n_{j}^{\text{obs}}$ observed values of $x_{j}^{\text{mis}}$ as the initial guess. After initialization, for each missing variable, the imputation model is decided using L1 penalty. For the selected imputation model, likelihood (or ridge) estimates $\hat{\beta }$ and $\hat{\Sigma }\left(\hat{\beta }\right)$. A random sample is drawn from this posterior distribution to obtain new updated value of $\hat{\beta }$ that will be used then to obtain the estimated values of missing observations. The resulting pseudo Algorithm 1 gives a detailed representation of the proposed MI technique to impute the missing values in continuous (normal) and binary variables.

**Algorithm 1** Pseudocode for the proposed algorithm

**Require: X** an *n* × *p* matrix, *M* number of required multiple imputations

 **for** *m* = 1, …, *M* **do**

  Make initial guess for missing values

  **while** not converged **do**

   **for** *j* = 1, …, *q* **do**

    Fit $x_{j}^{\text{mis}}∼X=\left(X^{\text{comp}},X_{-j}^{\text{mis}}\right)$ using L1 penalty;

    Obtain **X**^select^ comprising *p*_info_ informative predictors;

    Compute ridge estimates (or likelihood estimates) $\hat{\beta }$ and $\hat{\Sigma }\left(\hat{\beta }\right)$ from $x_{j}^{\text{mis}}∼X^{\text{select}}$;

    Obtain ${\hat{\beta }}_{\text{new}}=\text{a}\;\text{random}\;\text{sample}\;\text{from}\;\text{MVN}(\hat{\beta },\hat{\Sigma }\left(\hat{\beta }\right))$;

    Compute ${\hat{\eta }}_{\text{new}}=X_{n_{j}^{\text{mis}}\times p}\cdot {\hat{\beta }}_{\text{new}}$;

    **if** **x**_*j*_ is continuous **then**

     $x_{j}^{\text{mis}}$ ← update imputed values using ${\hat{\eta }}_{\text{new}}+\text{noise}$;

    **else if** **x**_*j*_ is binary **then**

     Draw a random vector **u**_*j*_ of size $n_{j}^{\text{mis}}$ from the uniform distribution on (0, 1);

$$\begin{array}{c} x_{j}^{\text{mis}}\leftarrow \text{update}\;\text{imputed}\;\text{values}\;\text{using}\{\begin{array}{cc} 1 & \text{if}\;\;u_{j}<{\left[1+\text{exp}\left(-{\hat{\eta }}_{\text{new}}\right)\right]}^{-1},\\ 0 & \text{otherwise}. \end{array} \end{array}$$

    **end if**

    **X** ← update using $x_{j}^{\text{mis}}$;

   **end for**

   **if** converged **then** $X_{m}^{\text{imp}}\leftarrow X$

   **end if**

  **end while**

 **end for**

In the algorithm, *p*_info_ represents the number of predictors selected for the imputation model using L1 penalty. Furthermore, $\hat{\Sigma }\left(\hat{\beta }\right)$ can be computed using Eq (1) for a GLM, whereas for a linear model it is obtained by the formula ${\chi^{2}\left(\nu \right)}^{-1}{\left(x_{j}^{\text{mis}}-X\hat{\beta }\right)}^{T}\left(x_{j}^{\text{mis}}-X\hat{\beta }\right)\cdot {\left(X^{T}X+\lambda I\right)}^{-1}$ for $\nu =n_{j}^{\text{obs}}-p_{\text{info}}$ degrees of freedom. The number of iterations required to get an imputed dataset depends on a convergence criterion. There is no hard and fast rule to decide the convergence in sequential regression imputation. A common practice to check the convergence is to plot mean and variance of the imputed values of different missing covariates against the iteration number. For convergence, these plots for *M* imputed datasets should be intermingled without any definite trend. Another option is to examine the relative magnitude of between sequence and within sequence variation. On convergence, the variance between different sequences should be smaller than the variance within each individual sequence. In general, misprHD meets these criteria after five to ten iterations. In a small scale simulation (the results of which are not shown here), we also investigated that how different methods of initialization of missing values affect the imputation process. In addition to *k*–nearest neighbours (*kNN*) imputation, we used two other approaches. First, the missing values of each variable were filled with a random sample drawn from the observed values of that variable. Second approach filled the missing values with mean or mode of the observed values of continuous or categorical variable respectively. These two approaches also converged between five to ten iterations. Although the quality of imputed value was little bit poor with initialization based on mean imputation but in general, there was not so much difference in the quality of imputed values based on different initialization approaches.

### 3.3 Selection and fitting of the imputation model

This Section describes imputation models considered with different choices of number of predictors, and the procedure followed to estimate the regression coefficients, i.e., penalized or unpenalized. We used the following imputation models, for each $x_{j}^{\text{mis}}$.

$$\begin{array}{ccc} \text{Model} & p_{\text{info}} & \text{Fitting Method}\\ \text{MI1:} & \text{decided with optimal value of L1 penalty} & \text{MLE}\\ \text{MI2:} & 0.5\times n_{j}^{\text{obs}} & \text{MLE}\\ \text{MI3:} & 0.8\times n_{j}^{\text{obs}} & \text{MLE}\\ \text{MI4:} & \text{decided with optimal value of L1 penalty} & \text{Ridge}\\ \text{MI5:} & 0.5\times n_{j}^{\text{obs}} & \text{Ridge}\\ \text{MI6:} & 0.8\times n_{j}^{\text{obs}} & \text{Ridge} \end{array}$$

For the model MI1 (and MI4), a 5-fold cross-validation was used to decide the optimal value of L1 penalty independently in the imputation model for each $x_{j}^{\text{mis}}$. The model was then fitted using MLE or ridge penalty, with the selected informative predictors. For MI2 (and MI5) and MI3 (and MI6) models, the value of L1 penalty was chosen so that it allowed us to select the desired number of informative predictors *p*_info_ for a particular imputation model. The standard MI software use likelihood estimation for fitting the imputation model. However, as *p* → *n* the likelihood estimates may become ustable with infinitely large standard errors and do not exist for *p* > *n*. In such case, ridge estimation can resolve the issue by providing unique estimates with better prediction, and a good compromise between variance and bias. To study the weakness of MLE in rich imputation model, we used both likelihood estimation and ridge estimation to obtain the posterior distribution of the parameter vector ***β*** given the observed data. The fitting of the imputation model with and without ridge penalty gave us an insight, how the imputations (based on LM or GLM) suffer and affect the inferences, while fitting a rich model with more predictors. For fitting the imputation models MI4, MI5, and MI6, the optimal value of L2 penalty was found independently for each $x_{j}^{\text{mis}}$ as response using 5-fold cross-validation.

## 4 Simulation study

A simulation study was designed to compare the performance of our proposed algorithm misprHD with the existing MI techniques. The performance was examined for a regression situation at the analysis stage, i.e., fitting a linear regression model with imputed datasets.

### 4.1 Simulation settings

We used two different scenarios in our simulation study with a focus on two types of variables i.e., continuous and binary variables.

**p > n with few informative predictors**: In this setting, we considered *p* = 50, 200, 500 predictors. The number of informative predictor (selected at random) was six and ten (each with *β*_*j*_ = 1) for *p* = 50 and *p* = 200, 500 respectively.

**n > p with all informative predictors**: In this setting, we considered *p* = 30, 60 predictors. All the predictors had non-zero regression coefficients. The values considered for the true regression coefficients were randomly drawn from a normal distribution with mean 1 and variance 1, and the value of the intercept was 1.

In each setting, *S* = 200 datasets were generated with a sample size *n* = 100. The covariates were drawn from a *p*–dimensional multivariate normal distribution with zero mean, unit variance, and an AR(1) correlation among covariates with *ρ* = 0.8. For *n* > *p* case, *ρ* = 0.5 was also considered to examine how the severity of collinearity affects the quality of imputations and the analysis of such imputed data. The response variable **y** in the complete data was generated using the linear model

y=Xβ+c·u,u∼N(0,1).

The value of *c* was chosen to have a signal-to-noise ratio equal to 3.0.

In each sample, half of the covariates chosen randomly were converted to binary using the percentile *P*_*j*∈[20,80]_ as threshold. In each simulated data, 10, 20, and 30% values were artificially deleted at random in 10 predictors (half of them were binary) chosen at random using the logit function

$$\begin{array}{c} \text{logit}\left[\text{Pr}\left(R_{ij}=0\;|\;x_{k},y\right)\right]=\gamma_{0}+\gamma_{1}y+\gamma_{k}x_{k}\;\;\text{for}\;j\neq k. \end{array}$$ (2)

The indicator *R*_*ij*_ = 0, if *i*th observation of **x**_*j*_ is missing, and *R*_*ij*_ = 1 otherwise. For each data, $x_{k}\in X^{\text{comp}}$ was randomly selected for each $x_{j\;\in \{1,\ldots ,q\}}^{\text{mis}}$. The value of *γ*_0_ was tuned corresponding to the fixed value of 0.5 for both *γ*_1_ and *γ*_*k*_ to achieve the approximate desired missing percentage in each $x_{j}^{\text{mis}}.$

### 4.2 Comparison criteria

The performance of the proposed technique is compared with mice [6], VIM [32], and MICE-DURR [14]. The basic building block of mice and VIM is a GLM that essentially requires *n* ≥ *p*. For high-dimensional data, both techniques use a selected number of predictors for each imputation model to have *n* ≥ *p*. mice focuses on removing the linearly dependent predictors using an iterative procedure which makes it much time consuming with increasing number of predictors. For *p* > *n*, VIM does not focus on the selection of informative predictors but always attempts to fit a LM or GLM with the first *n* predictors in any imputation model, and is therefore not so much costly in terms of processing time. MICE-DURR couples bootstrapping with regularization to impute missing values in high-dimensional settings. For regularization, it has the option to use lasso, adaptive lasso, or elastic net with a fixed value of 0.5 as elastic net mixing parameter. Our approach MI1 is similar to the imputation procedure MICE-IURR given by Zhao and Long, except that (a) if the optimal value of L1 penalty suggested an intercept model for the imputation, we used a smaller L1 penalty to fit a model with minimum number of predictors, and (b) a ridge regression was used to fit the imputation model if MLE faced some convergence issues. In our simulation study, with a relaxed lasso penalty (in our case MI6), we kept a record of variables included in the imputation model. It was observed all the times that all informative predictors were in the model. Also, this selection in each imputation model was compared with the selection at analysis stage. Every time, the selected variables at analysis stage were nested in our chosen rich imputation model making it semi-compatible that will lead to unbiased and consistent estimates.

The performance of the proposed algorithm was evaluated at imputation stage and the analysis stage. For imputation stage, the accuracy of the imputed values was measured with the help of Mean Squared Imputation Error (MSIE) computed as:

$$\begin{array}{c} \text{MSIE}=\frac{1}{S}\sum_{s}^{}[\frac{1}{M}\sum_{m}^{}\sum_{j}^{}\sum_{i=1}^{m_{j}}{\left(x_{ijm}^{*}-x_{ij}\right)}^{2}], \end{array}$$ (3)

where $x_{ijm}^{*}$ denotes the *i*th imputed value of the *j*th covariate in the *m*th imputed dataset corresponding to the observed value *x*_*ij*_ in the complete dataset. After imputing the missing values with different MI algorithms, the imputed datasets were used to fit the analysis model. Mean Squared Error (MSE($\hat{\beta }$)) computed from the analysis model was used as the performance measure. For *n* ≥ *p*, a linear model (LM) was fitted with each imputed data to obtain MSE($\hat{\beta }$). For *n* < *p* settings, lasso (least absolute shrinkage and selection operator) regression was used to obtain parameter estimates. For lasso regression, 10–fold cross-validation was used to compute the optimal value of L1 penalty. The estimated parameter vectors ${\hat{\beta }}_{m}\;\left(m=1,2,\ldots ,M\right)$ computed for each of the *M* = 5 imputed datasets were then combined using Rubin’s rules [1] to get MI estimates of ***β*** as $\hat{\beta }=M^{-1}\sum_{m=1}^{M}{\hat{\beta }}_{m}$. For $\hat{\beta }$ (that can be a likelihood or ridge estimate of ***β***), the covariance matrix can be computed as $\hat{\Sigma }=\text{cov}\left(\hat{\beta }\right)=W+\left(1+M^{-1}\right)\cdot B$, where $W=M^{-1}\sum_{m=1}^{M}\text{cov}\left({\hat{\beta }}_{m}\right)$, and $B={\left(M-1\right)}^{-1}\sum_{m=1}^{M}\left({\hat{\beta }}_{m}-\hat{\beta }\right){\left({\hat{\beta }}_{m}-\hat{\beta }\right)}^{T}$.

In simulation settings with *S* simulated samples, we computed $\text{MSE}\left(\hat{\beta }\right)=S^{-1}\sum_{s=1}^{S}{\left({\hat{\beta }}_{s}-\beta \right)}^{T}\left({\hat{\beta }}_{s}-\beta \right)$ with its split into variance and bias components given by $\text{var}\left(\hat{\beta }\right)=S^{-1}\sum_{s=1}^{S}{\left({\hat{\beta }}_{s}-\bar{\hat{\beta }}\right)}^{T}\left({\hat{\beta }}_{s}-\bar{\hat{\beta }}\right)\;\text{with}\;\;\bar{\hat{\beta }}=S^{-1}\sum_{s=1}^{S}{\hat{\beta }}_{s}$, and ${\text{Bias}}^{2}\left(\hat{\beta }\right)={\left(\bar{\hat{\beta }}-\beta \right)}^{T}\left(\bar{\hat{\beta }}-\beta \right)$ respectively.

### 4.3 Results

**Fig 1:**
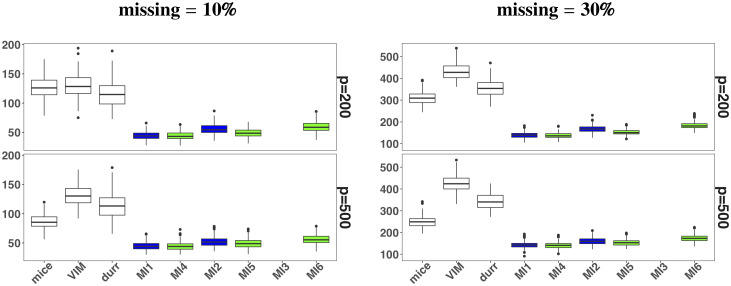
Simulation study: Box plots of MSIE. White boxes represent the threshold MI methods i.e., mice and VIM and durr. Blue and green boxes represent the MLE fit and ridge fit to the selected imputation model respectively.

The results of imputation error are given in Table 1. The results showed that if we develop a rich imputation model, the fitting of such model becomes problematic with increasing number of predictor e.g., for *p* = 200 & 500 with a sample size *n* = 100. Our proposal of using ridge penalty to fit the selected rich imputation model worked very well and provided good results not only for large number of predictors where MLEs did not exist, but also for *p* < *n* case. ML fit to the imputation model caused an increase in the imputation error as number of predictor was increased. On the other hand, ridge fit to the imputation model provided smaller imputation error. Some interesting results are also shown in Fig 1 in terms of Box plots. The white boxes are for three threshold methods (mice, VIM and durr), blue boxes are related to the imputation error obtained from ML fit to the imputation model, and green boxes are corresponding to the error based on a ridge fit. The thresholds showed poor performance in terms of imputation error. The boxplots showed the superiority of the proposed approach in terms of low imputation error. It even worked well for rich imputation model (MI6) where imputation error was unavailable due to non-existence of MLEs (MI3) in high-dimensional setting.

**Table 1:** Simulation study: Mean Squared Imputation Error (MSIE).

					misprHD (Imputation model fitting method)					
					MLE	Ridge	MLE	Ridge	MLE	Ridge
p	miss%	mice	VIM	durr	MI1	MI4	MI2	MI5	MI3	MI6
50	10	73.72	69.93	123.95	45.87	45.89	65.52	52.38	69.14	53.89
	20	163.31	139.75	248.27	94.8	94.02	131.2	105.59	154.23	112.87
	30	283.89	211.09	364.86	145.43	143.98	194.2	158.82	265.55	181.44
200	10	127.45	129.83	115.71	44.28	43.91	55.48	48.93	-	59.65
	20	229.27	274.68	235.88	91.53	90.06	112.06	100.48	-	121.18
	30	311.01	429.53	354.47	139.23	137.67	168.09	152.47	-	183.51
500	10	86.8	131.26	113.9	45.08	44.58	52.38	49.36	-	56.24
	20	169.91	274.23	231.59	93.3	92.45	106.78	100.98	-	114.96
	30	249	424.35	343.41	142.68	140.73	160.48	153.04	-	173.99
Results for *p* = 30, 60 with *ρ* = 0.8, when all predictors are informative i.e., *β*_*j*_ ≠ 0, ∀*j*.										
30	10	59.74	73.86	139.53	50.86	50.98	59.24	53.88	59.24	53.88
	20	129.21	159.5	268.24	104.35	104.28	126.9	112.45	126.9	112.45
	30	207.76	258.92	400.2	163.34	162.61	203.48	178.65	203.48	178.65
60	10	88.83	80.23	135.95	47.33	46.99	64.47	53.15	81.78	59.46
	20	210.88	161.02	259.43	96.71	96.3	130.04	107.02	194.23	132.38
	30	402.2	248.81	382.58	149.07	147.1	194.41	163.6	-	179.47
Results for *p* = 30, 60 with *ρ* = 0.5, when all predictors are informative i.e., *β*_*j*_ ≠ 0, ∀*j*.										
30	10	100.77	95.88	121.26	91.86	91.41	99.69	101.23	100.37	101.7
	20	210.22	192.5	242.44	187.13	187.95	210.75	210.92	210.33	211
	30	330.6	290.03	362.39	289.66	286.57	333.75	336.99	334.01	335.24
60	10	136.3	111.71	115.46	92.12	91.36	117.23	110.2	137.23	131.75
	20	302.19	213.12	231.57	186.56	184.87	232.53	216.71	299.13	279.77
	30	523.83	298.49	348.13	282.69	280.09	342.4	322.41	438.96	415.76

The performance of resulting imputations was also compared at analysis stage. To do so, we computed $\text{MSE}(\hat{\beta })$. The results of $\text{MSE}(\hat{\beta })$ for all simulation settings are given in Table 2. We could not obtain the imputed data with model MI3 for most samples in *p* > *n* settings. The imputation model faced convergence issues with GLM fit for large number of predictors relative to the sample size. The same problem was faced with *p* = 60 for 30% missing values. The MSEs for these settings are not given. For *p* = 30, the sample size was sufficiently large with respect to the number of predictors. For *p* = 30, MI2 and MI3 were same because the lasso penalty chose all 30 predictors in both models. As a result, we obtained identical results of MSEs for MI2 and MI3. Same case was also observed for MI5 and MI6 because of using the same imputed datasets. On the other hand, for high-dimensional case of *p* = 50, 200, 500, where only few predictors were relevant, a lasso regression was fitted to each imputed data independently. The regression coefficients so obtained were then combined using Rubin’s rules to obtain Mean Squared Error. For *p* = 30, 60, where all predictors were declared informative with non-zero parameter values, a linear regression was fitted to the imputed datasets.

**Table 2:** Simulation study: Results of MSE($\hat{\beta }$).

					misprHD (Imputation model fitting method)					
					MLE	Ridge	MLE	Ridge	MLE	Ridge
p	miss%	mice	VIM	durr	MI1	MI4	MI2	MI5	MI3	MI6
50	10	1.87	2.2	2.3	1.7	1.7	1.82	1.71	1.84	1.73
	20	2.35	2.96	2.82	1.81	1.82	2.12	1.84	2.32	1.86
	30	3.79	5.96	3.1	1.97	1.96	2.41	2.01	3.86	2.06
200	10	6.2	6.6	6.43	5.85	5.89	5.95	5.88	-	5.87
	20	6.45	7.26	6.87	5.98	5.96	6.08	5.94	-	5.97
	30	6.78	7.87	7.19	6.27	6.26	6.29	6.22	-	6.24
500	10	6.56	6.95	6.83	6.22	6.23	6.29	6.28	-	6.36
	20	6.99	7.33	7.21	6.45	6.45	6.5	6.44	-	6.46
	30	7.19	7.88	7.46	6.64	6.59	6.64	6.59	-	6.65
Results for *p* = 30, 60, when *ρ* = 0.8 and all predictors are informative i.e., *β*_*j*_ ≠ 0, ∀*j*.										
30	10	181.42	210.11	184.48	164.99	163.33	176.19	159.73	176.19	159.73
	20	192.73	238.74	182.46	163.12	159.34	181.66	156.93	181.66	156.93
	30	208.73	275.4	181.47	162.24	160.66	189.74	151.49	189.74	151.49
60	10	835.56	859.77	726.65	732.99	724.37	804.63	717.01	841.87	704.99
	20	1020.01	1042.21	711.25	717.04	704.13	817.36	679.49	1024.78	633.56
	30	1221.6	1327.28	705.15	690.36	679.86	819.15	652.56	-	626.07
Results for *p* = 30, 60, when *ρ* = 0.5 and all predictors are informative i.e., *β*_*j*_ ≠ 0, ∀*j*.										
30	10	14.99	15.81	14.49	13.02	12.75	14.49	12.68	14.49	12.68
	20	18.04	20.51	16.53	14.09	13.64	16.29	13.25	16.29	13.25
	30	22.08	29.8	18.23	15.68	14.7	18.87	14.03	18.87	14.03
60	10	95.24	100.65	87.25	84.04	83.74	87.07	80.95	93.89	79.59
	20	120.72	133.98	91.95	86.95	86.35	94.87	79.7	118.29	75.24
	30	150.9	200.57	95.2	88	85.47	97.94	81.28	127.56	74.54

**Fig 2:**
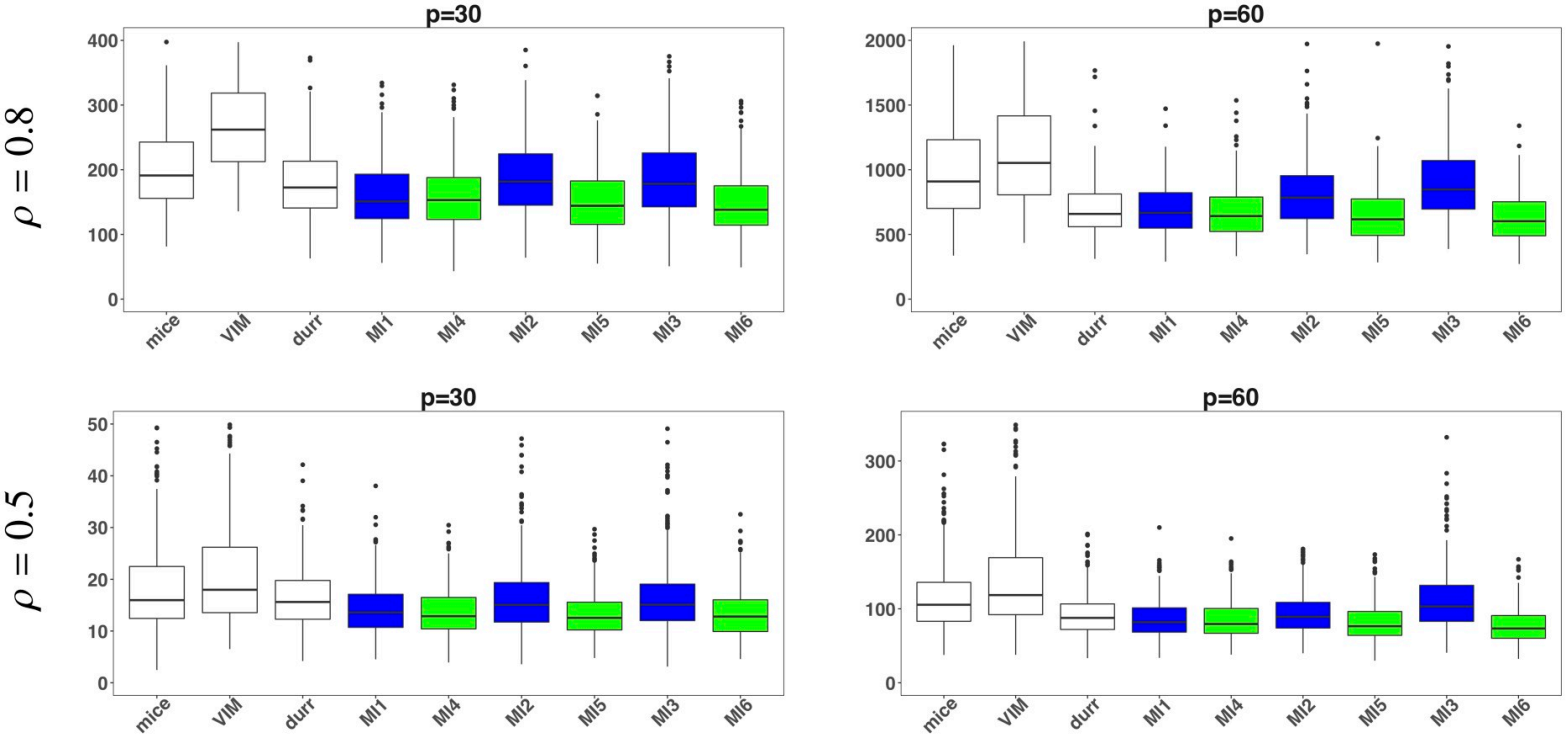
Simulation study: Box plots of MSE($\hat{\beta }$) for 30% missing values. White boxes represent the threshold MI methods i.e., mice and VIM and durr. Blue and green boxes represent the results based on imputed data using the MLE fit and ridge fit to the selected imputation model respectively.

The results exhibited that the idea of fitting a rich imputation model with ridge penalty provided better results than the other MI techniques, especially for high-dimensional data. The results of imputation model with likelihood fit (MI1) and with ridge fit (MI4) were approximately same. It is due to the sufficiently large sample than the number of predictors which were selected using the optimal value of L1 penalty. The ridge penalty chosen, to fit the model MI4, on the basis of 5-fold cross-validation was almost zero. MICE-DURR also uses L1 penalty with bootstrap samples but its overall performance was poorer than others. For *p* = 50, 200, 500, all MSEs are less than 10 because only few predictors were declared informative with value 1 for each true parameter. The results with low values of MSEs also reflect that the selection of relevant variables worked well with imputed datasets obtained from all MI algorithms. In case of all informative predictors, although the proposed approach was much better than the existing ones but MSEs were much higher for *ρ* = 0.8 than those obtained for *ρ* = 0.5. This raise was not the impact of imputations but the cause was fitting of linear model with high multicollinearity. The results of MSE($\hat{\beta }$) of substantive models for 30% missing values with *p* = 30 & 60, based on likelihood estimates are also given in terms of box plots in Fig 2. The white boxes represent threshold methods, whereas, blue and green boxes are linked with imputed data obtained using ML fit and ridge fit to the imputation model respectively. The threshold methods showed a higher MSEs followed by MSEs associated with MLE based imputed data. The MSEs associated with imputed data based on L2 penalty were minimum reflecting that the use of ridge penalty at imputation stage was also effective at the analysis stage.

### 4.4 Computational speed

The proposed algorithm is also time efficient than the standard MI software tools for high-dimensional settings. For imputing the missing values with all considered approaches, we used 2.20 GHz Intel(R) Xeon(R) E5-4620 CPU and noted the time required by different algorithms to impute 10% missing values in each of 200 simulated datasets. The average over these 200 time values (in seconds) is considered. The resulting average time required to impute one dataset, in different simulation settings, is given in Table 3. The time to impute missing values required by mice rapidly increases with increasing number of predictors and is not a good choice for high-dimensional data settings. VIM although takes less time for imputation because it is not performing any kind of variable selection and also ignores the uncertainty factor that is the basic concept of MI. The effect of such deficiency can be seen in the form of poor results of the analysis based on these imputations. The proposed approach is a better option with respect to processing time also for imputing the missing data especially in high-dimensional data structures.

**Table 3:** Average (of *S* = 200 elapsed time values) processing time (in seconds) required by different algorithms to impute one dataset with 10% missing values.

				misprHD (Imputation model fitting method)					
				MLE			Ridge		
p	mice	VIM	durr	MI1	MI2	MI3	MI4	MI5	MI6
50	12.02	6.61	255.25	72.23	128.94	130.28	118.76	149.47	150.34
200	822.99	28.99	213.02	99.06	179.90	-	138.76	198.21	187.06
500	24016.82	123.24	140.40	144.27	221.05	-	168.06	209.66	243.42

## 5 Application

We considered a real dataset from UCI machine learning repository [33] to compare the performance of our proposed approach with the existing MI approaches considered so far. The data is about the diagnoses of cardiac Single Proton Emission Computed Tomography (SPECT) images. *n* = 80 patients were diagnosed as normal or abnormal on the basis of *p* = 44 predictors. The data can be accessed at http://archive.ics.uci.edu/ml/datasets/SPECTF+Heart. The likelihood estimates did not converge for the data, so the ridge estimates were considered as true parameters and *S* = 100 binary responses were simulated. The optimal value of ridge penalty was decided using leave-one-out cross-validation. Like simulation study, 10%, 20%, and 30% values were missed at random artificially in half predictors (i.e., 22 predictors) using the logit function given in Eq (2). The predictors for missing values were selected randomly and independently in each of *S* = 100 samples. The likelihood estimates were not converging with the imputed data also and a ridge regression was fitted to compute MSEs. An independent 10-fold cross-validation was used for each imputed data to decide the optimal value of ridge penalty. The results of MSE for the ridge estimates, averaged over 100 samples are given in Table 4. Since the ridge estimates are biased, the resulting MSEs were further split into variance and bias (using the formulas given in Section 4.2) components to observe the contribution of bias in MSEs. VIM showed the poorest results of MSE among all imputation approaches considered. mice was better than VIM but its performance was poorer than others. The analysis of imputed data based on the most rich imputation model (fitted with ridge penalty) exhibited the best performance with the lowest value of MSE. However, the contribution of bias in the MSE was higher than others. We are not considering the individual parameter estimates and their CI coverage for this real data. The standard errors of ridge estimates are not so meaningful because of bias. A confidence statement using such SE ignoring the bias would be misleading.

**Table 4:** Heart data results of MSE with its split into variance and bias components. The results are obtained when a ridge regression was fitted to the data imputed with all proposed and existing imputation methods.

	10% missing			20% missing			30% missing		
Method	MSE	var	bias^2^	MSE	var	bias^2^	MSE	var	bias^2^
mice	0.68	0.56	0.12	0.77	0.59	0.18	0.87	0.61	0.26
VIM	0.81	0.69	0.12	0.91	0.75	0.15	1.08	0.88	0.21
durr	0.68	0.50	0.19	0.74	0.43	0.31	0.84	0.35	0.49
MI1	0.65	0.53	0.11	0.71	0.54	0.17	0.81	0.58	0.23
MI2	0.68	0.56	0.12	0.81	0.65	0.16	1.05	0.83	0.23
MI3	0.65	0.52	0.13	0.74	0.56	0.18	0.93	0.68	0.25
MI4	0.63	0.50	0.13	0.67	0.48	0.19	0.73	0.48	0.25
MI5	0.58	0.42	0.16	0.62	0.38	0.24	0.67	0.36	0.31
MI6	0.56	0.39	0.17	0.59	0.30	0.29	0.66	0.26	0.40

## 6 Discussion

In biomedical research, the researchers often face high-dimensional data for the analysis. Missing values are also common in such data. Analyzing the data without appropriate handling of missing values will cause biased inferences. Multiple Imputation (MI) is a flexible and the most popular approach to handle missing data. However, many practical issues arise regarding selection of variables for the imputation model in high-dimensional data. In the literature ([1, 8, 11, 12, 25, 34, 35]), it is discussed with theoretical and empirical evidence that if the imputed complete variables used to draw the inferences were not included in the imputation model, then the correlations between the imputed variables and the variables failed to be part of the imputation model will be biased towards zero. For high-dimensional data, such situation is common to occur. In this paper, our focus was to propose a course of action in high-dimensional data to select an imputation model based on all those variables which will be used in the inferences from the imputed complete data. For *p* ≫ *n*, some selection can be performed using the formal variable selection procedures. But there is no guarantee that the same selection will be used in the analysis model. Such incompatibility can cause biased estimates. It is difficult to attain compatibility with surety, especially when the imputer and the analyst are selecting their models independently. An effort can be made to avoid bias by making the imputation model semi-compatible. Semi-compatibility can be achieved by nesting the analysis model in the imputation model. This is only possible by including as many predictors as possible in the imputation model [8] using some formal variable selection technique. According to Liu et al. [36] recommendations, while building the imputation model, the first priority of the imputer should be to make an effort to have a compatible model, otherwise focus should be on improving the prediction quality of the imputation model. We used L1 penalty to do so, and instead of using its optimal value we used the value of L1 penalty that allowed us to select a large number of predictors (with maximum of *n* predictors). The second major issue is to obtain the posterior distribution of the parameters. The information about the covariance matrix for lasso estimates will be missing because of the nature of penalization. The GLM fit to such a big model usually results in unstable estimates with large SE. To overcome this problem, we proposed to use ridge regression for fitting the imputation model.
